# Sleep Satisfaction May Modify the Association between Metabolic Syndrome and BMI, Respectively, and Occupational Stress in Japanese Office Workers

**DOI:** 10.3390/ijerph19095095

**Published:** 2022-04-22

**Authors:** Helena Pham, Thomas Svensson, Ung-il Chung, Akiko Kishi Svensson

**Affiliations:** 1 Precision Health, Department of Bioengineering, Graduate School of Engineering, The University of Tokyo, 7-3-1 Hongo, Bunkyo-ku 113-8656, Tokyo, Japan; helena.ptl00@gmail.com (H.P.); helixcm1@g.ecc.u-tokyo.ac.jp (U.-i.C.); kishi@bioeng.t.u-tokyo.ac.jp (A.K.S.); 2 Department of Clinical Sciences, Lund University, Skåne University Hospital, 20502 Malmö, Sweden; 3 School of Health Innovation, Kanagawa University of Human Services Graduate School, Research Gate Building Tonomachi 2-A 2, 3F, 3-25-10 Tonomachi, Kawasaki-ku, Kawasaki-shi 210-0821, Kanagawa, Japan; 4 Clinical Biotechnology, Center for Disease Biology and Integrative Medicine, Graduate School of Medicine, The University of Tokyo, 7-3-1 Hongo, Bunkyo-ku 113-8655, Tokyo, Japan; 5 Department of Diabetes and Metabolic Diseases, The University of Tokyo, 7-3-1 Hongo, Bunkyo-ku 113-0033, Tokyo, Japan

**Keywords:** the Brief Job Stress Questionnaire, psychological stress, occupational stress, obesity, risk factor, occupational cohort, metabolic syndrome

## Abstract

The association between obesity and psychological stress is ambiguous. The aim is to investigate the association between metabolic syndrome (MetS) and body mass index (BMI), respectively, with occupational stress among Japanese office workers. The study is a secondary analysis of the intervention group from a randomized controlled trial. There are 167 participants included in the analysis. Occupational stress is self-reported using the Brief Job Stress Questionnaire (BJSQ). BMI and the classification of MetS/pre-MetS was based on the participants’ annual health check-up data. The primary exposure is divided into three groups: no MetS, pre-MetS, and MetS in accordance with Japanese guidelines. The secondary exposure, BMI, remains as a continuous variable. Multiple linear regression is implemented. Sensitivity analyses are stratified by sleep satisfaction. Pre-MetS is significantly associated with occupational stress (7.84 points; 95% CI: 0.17, 15.51). Among participants with low sleep satisfaction, pre-MetS (14.09 points; 95% CI: 1.71, 26.48), MetS (14.72 points; 95% CI: 0.93, 28.51), and BMI (2.54 points; 95% CI: 0.05, 4.99) are all significantly associated with occupational stress. No significant associations are observed in participants with high sleep satisfaction. The findings of this study indicate that sleep satisfaction may modify the association between MetS and BMI, respectively, and occupational stress.

## 1. Introduction

Metabolic syndrome (MetS) is a multifactorial group of risk factors strongly associated with the development of cardiovascular diseases (CVDs) and type 2 diabetes mellitus (T2DM) [1]. The diagnosis of MetS is characterized by physiological and biochemical abnormalities, including visceral obesity, hypertension, glucose intolerance, and atherogenic dyslipidemia [2]. Previous studies have suggested that these risk factors interact synergistically and together increase the risk of morbidity and mortality from CVD. Furthermore, myocardial infarction and stroke are three times more prevalent in those with MetS compared to those without the syndrome [3]. With a worldwide prevalence of approximately 25%, MetS appears to become more common with the concurrent increase in obesity, overnutrition, and spread of Western lifestyle [4]. In order to combat this growing concern, early diagnosis and prevention of MetS are important approaches in reducing the risks of CVDs. There is accordingly an urgent need to identify the modifiable risk factors of MetS as they provide useful information for primary preventions.

In Japan, CVD is one of the leading causes of death, and together with other lifestyle disorders, it has become a serious health issue, especially among the working population [5]. In order to decrease this burden, the Japanese Ministry of Health, Labour and Welfare (MHLW) introduced obligatory annual health checkups (AHC). The examinations include basic weight and height measurements, as well as blood tests for anemia, glucose, lipids, and liver function. Moreover, by focusing on visceral-fat obesity, nutrition, physical activities, and unhealthy habits, the system intends to identify those who suffer from or are at risk of lifestyle-related disorders such as MetS [6].

At the same time, occupational stress, as a result of global economic growth and work intensification, has become a major cause of overwork-related disorders [7]. Not only does it increase the risk of turnover [8] and long-term sickness absence [9], but it is also associated with longer working hours, which in turn leads to higher risks of morbidity and mortality [10]. In Japan, the incidents of karoshi (death by cerebrovascular and CVDs due to overwork) and karojisatsu (suicide due to overwork) have led to an urgent need to regulate these psychosocial work hazards. Due to the increasing cases of occupational stress, the MHLW established a stress check system in 2015, with the goal of preventing mental illness and improving work environments [11]. Application of the Brief Job Stress Questionnaire (BJSQ), a validated test used to measure occupational stress in Japan, enables early identification of highly stressed employees. This could in turn facilitate the initiation of preventive measures which may effectively reduce psychosocial distress among workers [12].

Another risk factor to be considered when it comes to the development of CVDs and T2DM is obesity. A metric that is used to identify obesity is body mass index (BMI), calculated as weight in kilograms by height in meters squared (kg/m^2^). BMI is currently not included in the Japanese criteria for MetS and its effectiveness in identifying obesity has been questioned as it does not discriminate fat mass from lean mass [13]. It is, however, unclear how much the method misclassifies individuals in larger groups because waist circumference (WC) and BMI are closely associated [14]. Nonetheless, BMI is an extensively validated metric of obesity and is still widely used today. It may therefore still be of interest to consider this variable when predicting the risk of CVDs.

The association between obesity and psychological stress is in turn ambiguous, as previous findings have been inconsistent and contradictory. Many studies have suggested a positive correlation between stress and BMI [15,16,17,18], while others have shown no significant association [19,20]. It is important to note that a majority of these studies have been conducted in Western countries. Results from Korea and China have instead shown an inverse association between stress and BMI [21,22]. Further understanding of this association may provide a deeper comprehension of the stress–metabolism relationship.

A number of studies have revealed a positive correlation between occupational stress and components of MetS, particularly obesity, dyslipidemia, and hypertension [23,24]. However, these results have not been entirely consistent, and the relationship has not been thoroughly studied among office workers. Stress may therefore be a direct or indirect risk factor of MetS and further knowledge of this association may be beneficial in establishing programs to reduce work stress and thereby the incidence of CVDs. Thus, this study aims to study the association between MetS and BMI, respectively, and occupational stress, using AHC data and questionnaires, among Japanese office workers.

## 2. Materials and Methods

### 2.1. Study Population

The participants of this study were formerly part of a 3-month randomized controlled trial (RCT), recruited from 5 different companies in Tokyo with over 1000 employees, respectively. Participants were full-time managerial, professional, or clerical workers. Participants were recruited from employees who had taken the AHC and who, based on their results, had been categorized with MetS or being at high risk of MetS. Concisely, 272 participants were enrolled in the RCT and among these, 181 were randomized for intervention whereas 91 were randomized to the control group (allocation ratio of 2:1), stratified according to gender and age. Two of the participants randomized to the intervention group declined to provide consent and were therefore excluded. Throughout the entire 3-month study period, the 179 eligible participants in the intervention group were asked to use a wearable device, the Fitbit Versa created by Fitbit Inc. (San Francisco, CA, USA), details at https://www.fitbit.com (accessed on 8 January 2022), together with its mobile application. The participants also completed the AHC, where data could be used to categorize the MetS status (MetS, at risk of MetS or without MetS) and calculate the BMI of each individual. The present study was therefore composed of a secondary analysis of the intervention group from the RCT.

**Figure 1:**
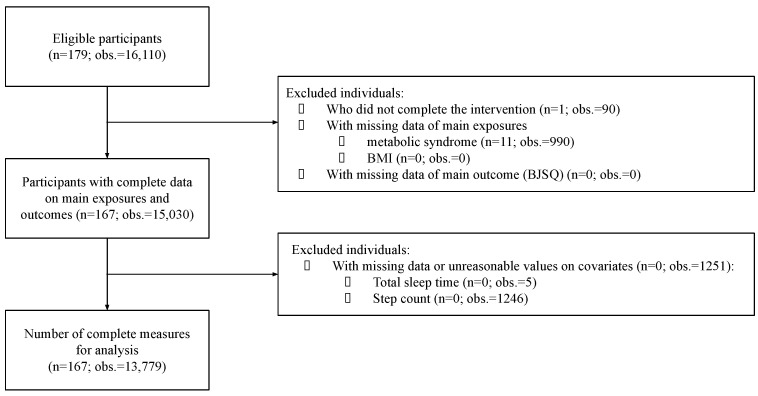
Flowchart illustrating the inclusion and exclusion of participants (*n*) and observations (obs.) in the present study.

For this particular study, 179 individuals with a total of 16,110 observations were eligible for analysis. Some participants may occasionally have had missing observations on daily measurements (e.g., if the wearable device was not worn or if a daily question was left unanswered) but they were still included in the analysis. Their inclusion was possible as the final analyses considered average values of repeated measures. Excluded participants were those who did not complete the intervention (*n* = 1; 90 observations) and those with missing data on the main exposures, MetS (*n* = 11; 990 observations), BMI (*n* = 0; 0 observations), or the main outcome, BJSQ (*n* = 0; 0 observations). Additional exclusions of observations were those with unreasonable values (daily step count < 1000: *n* = 0; 1246 observations) or missing data (immeasurable total sleep time: *n* = 0; 5 observations) for covariates (Figure 1). At last, 167 participants were included in the analyses.

### 2.2. Exposure Variables

The Japanese criteria defining MetS were established in 2005 and consider waist circumference (WC; ≥90 cm for women and ≥85 cm for men) as a compulsory criterion [25]. The remaining non-obligatory criteria include (1) hypertriglyceridemia (≥150  mg/dL) and/or low HDL-cholesterol (< 40  mg/dL), (2) high systolic (≥130 mmHg) and/or diastolic blood pressure (≥85 mmHg), and (3) elevated fasting glucose levels (≥110  mg/dL). At least 2 of these criteria together with a WC above cutoff value are required for the diagnosis of MetS. Those with a waist circumference above the cutoff value but meeting only one criterion for glucose, lipid, or blood pressure were considered as pre-MetS [26]. Those without measurements on WC could not be diagnosed and were subsequently excluded due to missing data. Ultimately, the main exposure of this study was divided into three categories: no MetS, pre-MetS, and MetS. BMI was calculated with data from the AHC and this exposure remained as a continuous variable in our analysis. The rationale for considering BMI as a continuous variable instead of categorizing participants according to their obesity status was absence of underweight individuals and the high proportion of obese study participants; the original RCT’s inclusion criteria focused on participants with MetS or who were at high risk of MetS.

### 2.3. Outcome Variable

The main outcome of this study was occupational stress, measured using the BJSQ, a validated test recommended by the Japanese Stress Check Program [27]. The participants answered the questionnaire at the onset of the 3-month study period. The BJSQ consists of 57 items used to assess a wide range of job stressors, stress responses, and social factors. In the present analysis, we summed the 4-point Likert scale which ranged from 1 to 4 (low stress to high stress), in order to calculate a total BJSQ score, concordant with the program manual [28]. Questions 1–7, 11–13, 15, and 18–20 were reverse-scored. Thus, a higher total score indicates a higher degree of job stress, and this variable remained continuous in our analysis. The reliability scores (Cronbach’s α coefficients) of the BJSQ’s three main subscales—job stressors, stress response, and buffering factors—were in the present study 0.80, 0.93, and 0.87, respectively.

### 2.4. Covariates

Information about sex and age (continuous) was obtained at the start of the study. Smoking status was classified into 4 categories at baseline: non-smoker, past-smoker, current smoker <20 cigarettes per day, and current smoker ≥20 cigarettes per day. Alcohol consumption was measured continuously throughout the entire study period; an average intake of ethanol (g/day) was calculated for each individual based on daily consumption (alcohol contents in brackets) of beer (5%), sake (5%), shochu (25%), chu-high (7%), cocktail (5%), wine (12%), whiskey (40%), and plum wine (15%). Average alcohol intake was categorized into low average daily intake (<20.0 g ethanol) and high average daily intake (≥20.0 g ethanol) in accordance with guidelines by the Japanese MHLW [29]. Daily intake of staple foods (i.e., rice, noodles), main dishes (i.e., eggs, meat, fish, soybeans), and dairy products (i.e., milk, cheese, yogurt) was measured as number of servings per day and an average number of servings was calculated for each food category. Daily step count and total sleep time was obtained using the wearable device whereas sleep satisfaction was obtained each morning using a single item from the St Mary’s Hospital Sleep Questionnaire [30]: “How satisfied were you with last night’s sleep?”. Answers were provided on a 5-point Likert scale (“1 = Very unsatisfied”, “2 = Moderately unsatisfied”, “3 = Slightly unsatisfied”, “4 = Fairly satisfied”, or “5 = Competely satisfied”). Each participant’s average daily values for step count (continuous per 100 steps), total sleep time (hours), and sleep satisfaction were calculated and used in the main analysis.

### 2.5. Statistical Analyses

In order to statistically analyze baseline characteristics, analysis of variance (ANOVA) test was implemented for numerical variables and chi-square test was used for categorical variables. The main analysis was based on multiple linear regression, used to study the association between MetS and BMI, respectively, and BJSQ. The main exposures were analyzed separately using three models with different adjustments of confounding variables: Model 1 was adjusted for sex and age. Model 2 was further adjusted for smoking status, average daily alcohol consumption, and average number of servings per day of staple foods, main dishes, and dairy products. Model 3 was further adjusted for average steps per day, average total sleep time, and average sleep satisfaction.

Post-hoc analyses were performed through sensitivity analyses by stratifying the main analyses according to sleep satisfaction, a possible modifier of the association between MetS, BMI, and occupational stress. A binary sleep satisfaction variable was created using the median value of average daily sleep satisfaction as the cutoff (low sleep satisfaction: <3.55; high sleep satisfaction ≥3.55). The *p*-value for the interaction between sleep satisfaction and main exposure was calculated using the likelihood ratio test where the final multivariable-adjusted model (Model 3) was compared to a model without this interaction.

All statistical analyses were performed using Stata Statistical Software version 16.1 (StataCorp LLC (College Station, TX, USA)). A 2-tailed *p* < 0.05 was considered to be statistically significant.

### 2.6. Ethical Considerations

The study was performed in accordance with relevant ethical guidelines and regulations. All participants received both written and verbal information about the study and its purpose. Each participant understood that participation was voluntary and that discontinuation was possible at any time regardless of cause, without subsequent penalty or disadvantage. All participants provided written informed consent, and the study was approved by the Ethical Committee of the School of Engineering, The University of Tokyo (approval number: KE18-44).

## 3. Results

### 3.1. Baseline Characteristics

The baseline characteristics based on the classification of MetS are presented in Table 1. The majority of the study participants (92.8%) were men (*p* = 0.001), and the largest proportion of women (17.3%) was observed in the no MetS group. Those diagnosed with MetS represented the oldest group, whereas those without MetS were 4.4 (±1.4) years younger and represented the youngest group (*p* = 0.008). Although not significant, the pre-MetS group had the highest prevalence of current smokers (<20 cigarettes/day), while the no MetS group had the highest prevalence of heavy smokers (≥20 cigarettes/day). The no MetS group was also more likely to consume more alcohol (≥20 g ethanol/day). Moreover, the no MetS group had the marginally lowest number of staple food servings, albeit non-significantly, whereas all three groups had the same number of servings of main dishes and dairy products. With marginal differences, individuals with MetS reported to have the shortest total sleep time, whereas the pre-MetS group had the lowest daily mean step count and sleep satisfaction.

### 3.2. Main Analyses

Individuals with pre-MetS had a positive association with occupational stress levels, and the largest effect size was observed in Model 1 (10.8 points; 95% CI: 2.85, 18.82) (Table 2). This association was significant in all three models; the inclusion of covariates slightly attenuated the association in Model 2 (9.02 points; 95% CI: 0.82, 17.22) and the association was further attenuated with the additional inclusion of covariates in the final multivariable model (Model 3: 7.84 points; 95% CI: 0.17, 15.51). There was no significant association between the diagnosis of MetS and occupational stress levels (Model 1: 3.3 points; 95% CI: −5.09, 11.69). This result did not change following the inclusion of additional covariates in Model 2 (0.71 points; 95% CI: −7.98, 9.40) or Model 3 (2.75 points; 95% CI: −5.42, 10.91).

BMI was not significantly associated with occupational stress levels (Model 1: 1.00 points; 95% CI: −0.40, 2.40) (Table 3). This association did not change following the inclusion of covariates in Model 2 (0.92 points; 95% CI: −0.50, 2.34) or Model 3 (1.32 points; 95% CI: −0.02, 2.65).

### 3.3. Sensitivity Analyses

Sensitivity analyses were stratified according to sleep satisfaction, with MetS (likelihood ratio test; *p* = 0.0535) and BMI (likelihood ratio test; *p* = 0.025) as exposures in separate statistical models. Among those with low sleep satisfaction, pre-MetS was positively and significantly associated with the BJSQ score in all models (Model 1: 16.36 points; 95% CI: 3.97, 28.75) (Table 4). The association was only slightly attenuated following the inclusion of additional covariates in Model 2 (14.29 points; 95% CI: 1.20, 27.37) and Model 3 (14.09 points; 95% CI: 1.71, 26.48). MetS was positively associated with the BJSQ score in Model 1 (12.42 points; 95% CI: −0.74, 25.57) and Model 2 (8.39 points; 95% CI: −5.81, 22.59; however, the association was statistically significant only in the final multivariable model (Model 3: 14.72 points; 95% CI: 0.93, 28.51). Similarly, among those with low sleep satisfaction, BMI was positively associated with the BJSQ score in Model 1 (2.12 points; 95% CI: −0.18, 4.42) and Model 2 (2.07 points; 95% CI: −0.44, 4.58); however, statistical significance was only observed in Model 3 (2.54 points; 95% CI: 0.05, 4.99). Among participants with high sleep satisfaction, neither pre-Mets, MetS, nor BMI were significantly associated with the BJSQ score (Table 5).

## 4. Discussion

The current study was a cross-sectional study conducted to investigate the association between MetS and BMI, respectively, and occupational stress among Japanese office workers. The main results show a positive association between pre-MetS and occupational stress scores using the BJSQ, indicating that individuals with pre-MetS tend to report higher stress levels in comparison to healthy individuals. This association was not significant among office workers who had been diagnosed with MetS. The main results also show that BMI is not significantly associated with occupational stress.

Previous studies have shown ambiguous results between occupational stress and components of MetS. However, contrary to our findings, the generally accepted view is that psychological stress is positively associated with the development of MetS [31]. The pathway is multifactorial, probably involving stress-induced changes in the hypothalamic-pituitary-adrenal (HPA) axis and the sympathetic nervous system (SNS) [32]. Beyond these physiological pathways, it has also been suggested that stress alters behaviors, perception of need, and personal control [33]. As a result, psychological stress has been closely linked to unhealthy behaviors that increase the risk of MetS [34]. Thus, in regard to the fact that this may be a contributive pathway to MetS, behavioral factors (e.g., smoking, physical inactivity) may be considered as mediators rather than confounding variables. Controlling for a mediator could in turn reduce or completely destruct an established relationship, preventing the possibility of finding a significant association between two variables [34].

In contrast to the MetS group, the pre-MetS group showed a positive and significant association with the BJSQ score. There may be two possible explanations for these findings. First, it is important to consider the effects of residual confounding factors. One such important factor that was not considered in our analysis is working hours. In Japan, most employees associate longer working hours with diligence and a strong sense of responsibility [35]. Moreover, male workers are generally much more subject to overtime work compared to women [36]. In the current study, all individuals categorized with pre-MetS were men, an observation which is thus of importance when interpreting the results. Secondly, depending on the AHC data and the number of individual risk factors, different health conditions indicate two different types of Specific Health Guidance (SHG), as part of the Japanese National Health Screening and Intervention Program for individuals above the age of 40 [37]. Both intensive health guidance and motivational health guidance offer initial counseling with a qualified healthcare professional (medical doctor, nurse, or dietician) where the participants are informed about their condition and instructed to set personalized behavioral goals. Individuals with MetS are classified as in need of intensive health guidance where they additionally receive active support for at least 3 months and a personalized follow-up consultation. However, individuals with pre-MetS are offered motivational health guidance, which does not include continuous support [38]. Those diagnosed with MetS may therefore acquire a higher level of health literacy and improvements in one lifestyle domain (e.g., diet or physical activity) may positively impact another, such as psychological stress.

The present study also included a sensitivity analysis that considered sleep satisfaction as a possible moderator for the association between MetS and BMI, respectively, and occupational stress. This post-hoc analysis was implemented as sleep satisfaction revealed a significant result in the main analysis. Data from prior research have suggested that sleep quality, which includes several dimensions of sleep, such as duration and feeling of restfulness, may influence MetS, BMI, and perceived stress [39,40,41]. One large longitudinal study on Japanese male workers reported that high-risk lifestyle parameters, including insufficient sleep, may be predictive of MetS onset, mainly through biological mechanisms and weight gain [42]. Sleep reactivity, i.e., the extent of which sleep disruption occurs due to stress exposure, is another element of sleep satisfaction where difficulty of falling and staying asleep are affected [43]. A recent cross-sectional study, which also implemented the BJSQ, concluded that sleep reactivity could affect occupational stress, specifically stress reaction [44]. Conversely, there is a consensus that stress disrupts sleep on multiple levels, including sleep efficiency and sleep depth [45]. With regard to previous research and the findings from the main analysis, a post-hoc analysis was adopted to gain a deeper understanding of sleep satisfaction as a possible moderator of the stress–metabolism relationship.

According to our results, both the pre-MetS group and the MetS group had a positive association with the BJSQ score if they also estimated low sleep satisfaction. Indeed, the likelihood ratio test resulted in a *p*-value of 0.0535, which is on the cut-off for being statistically significant. However, it is important to consider the wide 95% CI for both groups, as this may indicate that the statistical power is insufficient to demonstrate significant associations. These findings are nonetheless valuable to discuss as previous studies have shown that poor sleep quality can elevate the risk of obesity, dyslipidemia, hypertension, and insulin resistance and thereby lead to MetS and T2DM [46]. Although the definition of sleep quality varies among different studies, it mainly refers to subjective perception of sleep, such as sleep efficiency, restoration, and disturbance [47]. It is estimated that 50–60% of patients with MetS have obstructive sleep apnea (OSA), a clinical condition characterized by recurrent collapse of upper airways during sleep, which ultimately results in sleep fragmentation and intermittent hypoxia [48]. Not only does sleep deprivation lead to desynchronization of hormonal and metabolic regulations together with SNS and HPA axis activation [49] but recurrent hypoxia also exacerbates the cardiometabolic dysfunction through increased lipolysis, inflammation, and oxidative stress [50]. Thus, sleep quality may function as an important moderator in the stress–metabolism relationship and our observations highlight the need to consider this factor when predicting the risk of MetS.

The results of our main analysis showed that BMI as an independent exposure was not associated with occupational stress levels. However, in accordance with prior studies, a positive association was observed in those with low sleep satisfaction in the sensitivity analysis. Stress is known to disrupt sleep and insufficient sleep is in turn associated with higher weight and obesity [51]. This association is connected through several interacting pathways, incorporating cognitive behavior, physiology, and biochemistry. First, disrupted sleep results in decreased energy expenditure by decreasing thermogenesis and promoting fatigue, which in turn leads to increased sedentary behavior and reduced physical activity [52,53]. Second, shorter sleep duration generates more time to eat and it intensifies hunger for high-fat and high-carbohydrate foods, by altering the concentrations of appetite regulating hormones [54]. The combination of decreased leptin levels, and elevated ghrelin and cortisol levels, results in glucose intolerance and insulin resistance, which further aggravates the metabolic dysregulation [55]. Concordant with observations of MetS and occupational stress, sleep satisfaction may need to be considered as an important moderator for the association between increasing BMI and occupational stress.

The findings of our study may have important implications for both public health and clinical practice. Given the complex etiology of MetS, early identification and control of modifiable risk factors are crucial in order to prevent the increasing prevalence of CVDs and T2DM. Improvement of lifestyle is currently considered to be the most important method to combat MetS and obesity. To date, public health efforts, including the AHCs and SHG, have focused on programs to increase physical activity and improve the diet. However, along with these conventional targets, our observations support the need to address both sleep quality and occupational stress as part of a comprehensive treatment plan for high-risk individuals. Clinicians and health professionals may therefore increase efforts to promote healthy sleep and stress management. Similarly, an improved awareness of these parameters among employers and employees may provide a better understanding of current health guidelines, which in turn may effectively reduce risk factors for MetS in Japanese workers. Taken together, this study suggests the importance of integrating occupational stress and sleep into MetS policies. Longitudinal study designs are encouraged to establish causality and to elucidate the underlying mechanisms.

### Strengths and Limitations

The findings from this study should be interpreted in light of certain limitations. First, the statistical power may have been insufficient due to the small sample size. Second, considering the observational and cross-sectional nature of our analysis, there is no temporal aspect of the results which limits the possibility to establish a causal relationship. Hence, future studies with a larger number of participants and longitudinal analyses are highly warranted in order to elucidate the temporal associations between MetS, BMI, and occupational stress. Third, despite adjusting for highly relevant variables such as diet, sleep, and physical activity, there still may be residual confounders, owing to the highly multifactorial attributes of both MetS and stress. Fourth, the generalization of the results to other populations and countries should be done with caution, as the prevalence of MetS is highly dependent on the definition of its different components. Some systems focus on the accumulation of risk factors, whereas the Japanese government focuses on abdominal obesity [56]. Nevertheless, our findings may have important implications for companies and employers to develop early interventions and reduce occupational stress.

Despite these limitations, the study also has several strengths. First, we used validated definitions and criteria to assess both occupational stress and MetS, as recommended by the MHLW of Japan. The results are therefore highly relevant in the Japanese occupational context and may also be valuable for public health and clinical practice. Second, the consideration of BMI as a main exposure in separate analyses adds depth to the analyses and allows for the wider generalizability of our results. Third, the usage of mean values for repeated measures minimized the effects of missing values, thereby increasing the number of participants that were included in the analysis. Finally, the implementation of daily questionnaires and a wearable device allows for representative individual-level measures. Above all, the wearable device ensures continuous collection of data in an objective manner whereas questionnaires enable standardized measurements of daily habits.

## 5. Conclusions

The findings of this study show that there is a positive association between pre-MetS and occupational stress among Japanese office workers. In individuals diagnosed with MetS, this association was only observed if they also reported having low sleep satisfaction, suggesting that sleep quality may have a moderating effect. Similarly, BMI was only associated with occupational stress in those with low sleep satisfaction. Future studies with larger sample sizes using longitudinal analyses are encouraged to further investigate the temporal associations between MetS, increasing BMI, and occupational stress.

## Figures and Tables

**Table 1:** Baseline characteristics according to the classification of metabolic syndrome.

Characteristics	MetS *n =* 52	Pre-MetS *n =* 63	No MetS *n =* 52	*p* Value ^a^	Test Statistic
Men (%)	94.23	100	82.69	0.001	Chi2(2) = 13.02
Age at screening [mean (years ± SD)]	46.8 ± 6.6	43.3 ± 8.2	42.4 ± 7.3	0.008	F_2164_ = 5.04
Smoking status (%)				0.389	Chi2(6) = 6.31
Non-smoker	42.3	39.7	53.9		
Past smoker	32.7	33.3	21.2		
Current smoker (<20 cigarettes/day)	13.5	19.1	9.6		
Current smoker (≥20 cigarettes/day)	11.5	8.0	15.4		
Alcohol consumption (%)				0.198	Chi2(2) = 3.24
<20 g ethanol/day	53.9	42.9	36.5		
≥20 g ethanol/day	46.2	57.1	63.5		
Sleep satisfaction (5-point scale) [mean ± SD]	3.5 ± 0.4	3.4 ± 0.5	3.5 ± 0.4	0.157	F_2164_ = 1.87
Number of servings of staple foods [mean ± SD]	4.3 ± 1.0	4.4 ± 0.8	4.1 ± 0.9	0.453	F_2164_ = 0.79
Number of servings of main dish [mean ± SD]	4.2 ± 1.2	4.3 ± 1.0	4.3 ± 1.1	0.822	F_2164_ = 0.20
Number of servings of dairy product [mean ± SD]	1.9 ± 0.7	1.9 ± 0.7	1.9 ± 0.5	0.915	F_2164_ = 0.09
Number of steps/day [mean ± SD]	11,416 ± 3184	10,473 ± 2478	11,044 ± 1917	0.142	F_2164_ = 1.97
Total sleep (hours)/day [mean ± SD]	5.5 ± 0.6	5.6 ± 0.5	5.7 ± 0.5	0.264	F_2164_ = 1.34

Abbreviations: MetS: metabolic syndrome, SD: standard deviation ^a^ ANOVA test for age at screening and daily component variables; Chi-square test for categorical variables.

**Table 2:** Coefficients (β) and 95% confidence intervals for the association between metabolic syndrome and the Brief Job Stress Questionnaire score (*n* = 167).

The BJSQ	Model 1 ^a^	Model 2 ^b^	Model 3 ^c^
Explanatory variable	β (CI)	β (CI)	β (CI)
**Metabolic syndrome**			
**No MetS**	Reference	Reference	Reference
**Pre-MetS**	10.80 ** (2.85, 18.82)	9.02 ** (0.82, 17.22)	7.84 * (0.17, 15.51)
**MetS**	3.30 (−5.09, 11.69)	0.71 (−7.98, 9.40)	2.75 (−5.42, 10.91)
Intercept	121.47	117.43	163.24

Abbreviations: BJSQ: Brief Job Stress Questionnaire, CI: confidence interval, MetS: metabolic syndrome. Bold values denote statistically significant results. ^a^ Adjusted for sex and age. ^b^ Additionally adjusted for smoking, alcohol consumption, and average number of servings per day of staple foods, main dishes, and dairy products. ^c^ Additionally adjusted for average steps, sleep satisfaction, and sleep duration. * *p* < 0.05; ** *p* < 0.01.

**Table 3:** Coefficients (β) and 95% confidence intervals for the association between BMI and the Brief Job Stress Questionnaire score (*n* = 167).

The BJSQ	Model 1 ^a^	Model 2 ^b^	Model 3 ^c^
Explanatory variable	β (CI)	β (CI)	β (CI)
BMI	1.00 (−0.40, 2.40)	0.92 (−0.50, 2.34)	1.32 (−0.02, 2.65)
Intercept	100.16	95.93	128.37

Abbreviations: BJSQ: Brief Job Stress Questionnaire, CI: confidence interval, BMI: Body Mass Index. ^a^ Adjusted for sex and age. ^b^ Additionally adjusted for smoking, alcohol consumption, and average number of servings per day of staple foods, main dishes, and dairy products. ^c^ Additionally adjusted for average steps, sleep satisfaction, and sleep duration.

**Table 4:** The association between metabolic syndrome and body mass index, respectively, and the Brief Job Stress Questionnaire score in participants with low sleep satisfaction (*n* = 83).

The BJSQ	Model 1 ^a^	Model 2 ^b^	Model 3 ^c^
Explanatory variable	β (CI)	β (CI)	β (CI)
**Metabolic syndrome**			
**No MetS**	Reference	Reference	Reference
**Pre-MetS**	16.36 ** (3.97, 28.75)	14.29 * (1.20, 27.37)	14.09 * (1.71, 26.48)
**MetS**	12.42 (−0.74, 25.57)	8.39 (−5.81, 22.59)	14.72 * (0.93, 28.51)
Intercept	126.50	126.18	148.48
**BMI**	2.12 (−0.18, 4.42)	2.07 (−0.44, 4.58)	2.52 * (0.05, 4.99)
Intercept	76.22	78.58	77.07

Abbreviations: BJSQ: Brief Job Stress Questionnaire, CI: confidence interval, MetS: metabolic syndrome, BMI: Body Mass Index. Bold values denote statistically significant results. ^a^ Adjusted for sex and age. ^b^ Additionally adjusted for smoking, alcohol consumption, and average number of servings per day of staple foods, main dishes, and dairy products. ^c^ Additionally adjusted for average steps, sleep satisfaction, and sleep duration. * *p* < 0.05; ** *p* < 0.01

**Table 5:** The association between metabolic syndrome and body mass index, respectively, and the Brief Job Stress Questionnaire score in participants with high sleep satisfaction (*n* = 84).

The BJSQ	Model 1 ^a^	Model 2 ^b^	Model 3 ^c^
Explanatory variable	β (CI)	β (CI)	β (CI)
Metabolic syndrome			
No MetS	Reference	Reference	Reference
Pre-MetS	5.24 (−4.44, 14.93)	3.13 (−6.90, 13.15)	3.18 (−7.01, 13.37)
MetS	−6.98 (−17.1, 3.14)	−9.11 (−19.27, 1.05)	−8.14 (−18.72, 2.45)
Intercept	107.59	102.26	145.62
BMI	0.37 (−1.28, 2.02)	0.34 (−1.33, 2.01)	0.63 (−1.11, 2.37)
Intercept	102.52	96.92	147.90

Abbreviations: BJSQ: Brief Job Stress Questionnaire, CI: confidence interval, MetS: metabolic syndrome, BMI: Body Mass Index. ^a^ Adjusted for sex and age. ^b^ Additionally adjusted for smoking, alcohol consumption, and average number of servings per day of staple foods, main dishes, and dairy products. ^c^ Additionally adjusted for average steps, sleep satisfaction, and sleep duration.

## Data Availability

We cannot publicly provide individual data due to participant privacy in accordance with ethical guidelines. Additionally, the written informed consent we obtained from study participants does not include a provision for publicly sharing data. Qualifying researchers may apply to access a minimal dataset upon reasonable request by contacting the corresponding author.
